# Integrated gene expression analysis identifies shared inflammatory and metabolic pathways in polycystic ovarian syndrome, rheumatoid arthritis, and osteoarthritis

**DOI:** 10.5114/bta/214378

**Published:** 2025-12-17

**Authors:** Sri Chandana Mavulati, Sujatha Dodoala

**Affiliations:** 1 Department of Pharmacology, Shri Vishnu College of Pharmacy, Bhimavaram, Andhra Pradesh, India; 2 Department of Pharmacology, Institute of Pharmaceutical Technology, Sri Padmavathi Mahila Visvavidyalayam, Tirupati, Andhra Pradesh, India

**Keywords:** polycystic ovarian syndrome, inflammation, differentially expressed genes, gene ontology, encyclopedia of genes and genomes, pathway enrichment analysis

## Abstract

**Background:**

Polycystic ovary syndrome (PCOS) affects millions of women worldwide and is primarily known for its reproductive and hormonal symptoms. However, growing evidence suggests a strong link between PCOS and inflammation. Rheumatoid arthritis (RA) and osteoarthritis (OA) similarly involve systemic inflammation and immune dysregulation. Despite their distinct clinical manifestations, these disorders may share overlapping biological pathways. This study aimed to identify shared transcriptomic signatures between PCOS and autoimmune joint diseases such as RA and OA.

**Materials and methods:**

RNA sequencing datasets were downloaded from the publicly available Gene Expression Omnibus (GEO) database. After processing and quality filtering, a total of 73 samples from the GSE277906 and GSE89408 datasets were selected. DEG analysis was conducted using the DE-Seq2 package in RStudio by adjusting for a significant *p*-value < 0.1 and |log_2_ fold change| > 0.5. Gene Ontology (GO) and Kyoto Encyclopedia of Genes and Genomes (KEGG) analyses were performed to determine functional enrichment of genes and common pathways associated with the diseases.

**Results:**

A total of 10,492 and 9,892 DEGs were identified in PCOS vs. RA and PCOS vs. OA, respectively. Key genes dysregulated among the diseases included *TOMM34, DHCR24, CMAS, RBP1*, and *HSD3B2*, and the enrichment analysis revealed overlapping pathways involving immune regulation, mitochondrial dysfunction, oxidative stress, and proteasome activity. Notably, 201 GO pathways were shared by PCOS and OA, 123 by RA and OA, and 267 by PCOS and RA. All three conditions shared a set of 57 GO pathways, including mitophagy and ER stress.

**Conclusion:**

The identified common pathways signify the overlap between PCOS, RA, and OA. These findings support the hypothesis of systemic immunometabolic involvement in PCOS.

## Introduction

Polycystic ovary syndrome (PCOS) is a prevalent ovulatory, metabolic, and hormonal disturbance that affects 6–15% of women worldwide during their reproductive age (Joshi et al. 2014; Gupta et al. 2017; Skiba et al. 2018; Yu et al. 2023; Divya et al. 2024; Pallavi and Harshitha 2024). It is characterized by altered ovarian morphology, disturbances in menstrual history, and excessive androgen secretion (Joshi et al. 2014; Gupta et al. 2017; Skiba et al. 2018). Primarily considered a metabolic and reproductive condition, PCOS is increasingly recognized to be associated with significant comorbidities such as cardiovascular risk, insulin resistance, obesity, and certain cancers (Legro et al. 1999; Barry et al. 2014; Gilbert et al. 2018). In addition, recent studies have highlighted its association with inflammatory diseases, indicating that immune dysregulation may be involved in the pathogenesis of PCOS (Ghowsi et al. 2018; Sharmeen et al. 2021). Elevated levels of inflammatory markers such as tumor necrosis factor α (TNF-α), inter-leukin-6 (IL-6), and C-reactive protein (CRP) have been identified in women with PCOS, suggesting the possibility of immune dysregulation (Rudnicka et al. 2020, 2021).

Inflammatory conditions such as rheumatoid arthritis (RA) and osteoarthritis (OA) are two joint disorders with distinct etiologies and are highly prevalent in women. Clinical characteristics of these disorders include joint pain, inflammation, and tissue remodeling. RA is a chronic autoimmune disorder that affects the synovial tissue, causing inflammation and joint destruction (Bhakta 2025). In contrast, OA is regarded as a degenerative joint disorder caused by mechanical stress, cartilage breakdown, and aging (Moulin et al. 2025). However, recent findings suggest that OA is associated with inflammation, oxidative stress, and metabolic disturbances similar to those observed in women with PCOS (Courties et al. 2015; Robinson et al. 2016).

Emerging research has increased interest in inflammatory arthritis and its association with PCOS. Elevated levels of pro-inflammatory cytokines and shared risk factors such as obesity, insulin resistance, and disrupted lipid metabolism point toward common pathological profiles (Rudnicka et al. 2021; Kluzek et al. 2021). Additionally, research on differentially expressed genes (DEGs) and immune-related pathways has revealed associations between the molecular mechanisms of inflammatory diseases such as periodontitis and PCOS (Liu et al. 2024). These findings reinforce the hypothesis that PCOS involves inflammatory dysfunctions extending beyond the reproductive axis.

However, recent work in adolescents with PCOS has revealed reduced bone density and disrupted bone metabolism, indicating systemic involvement beyond reproductive function. Bone turnover markers such as Gla-OC (γ-carboxyglutamic acid-containing osteocalcin), Glu-OC (glutamate-containing osteocalcin), and CTX-I (C-terminal telopeptide of type I collagen) were found to be lower in obese adolescents with PCOS (Mizgier et al. 2025). Additionally, reduced levels of procollagen type I aminoterminal propeptide and CTX in women with PCOS indicate potential long-term skeletal health concerns even when bone mineral density appears normal (Lingaiah et al. 2017).

To gain a better understanding of potential overlapping pathogenic mechanisms, it is imperative to investigate the shared and unique genetic signatures associated with rheumatological and immune-mediated disorders in the context of PCOS. While numerous studies have focused on identifying key genes involved in PCOS, diabetes, and cancer, the overlapping mechanisms and inflammatory triggers in PCOS remain unclear (Bogari 2020; Hagenauer et al. 2024). Rapid advancements in genetic analysis have led to improved approaches for exploring the genetic and metabolic architecture of PCOS and inflammatory conditions. This study examines common pathways, DEGs, and shared ontologies among RA, OA, and PCOS. Additionally, gene expression microarray datasets were evaluated using publicly available data from the Gene Expression Omnibus (GEO) database.

The study focuses on investigating the molecular and immunological overlaps between reproductive, metabolic, and autoimmune diseases through gene mapping. Using tools such as Venn analysis and functional enrichment studies, we seek to identify common genes and signaling pathways across these conditions. The results provide insight into shared pathogenic mechanisms and help establish a framework for understanding immunometabolic interactions between reproductive and rheumatological disorders, thereby offering scientific support for developing advanced diagnostic and therapeutic strategies.

## Materials and methods

### Data collection and processing

GSE277906 and GSE89408 were retrieved from the publicly available GEO database (http://www.ncbi.nlm.nih.gov/geo) (Barrett et al. 2013). GSE277906 comprises mRNA-sequencing (RNA-seq) expression data of cumulus cells from 22 PCOS and 17 healthy control samples (Chen et al. 2024). For the OA and RA sequences, we used the GSE89408 dataset, from which 28 normal, 28 RA, and 22 OA samples were included in the current study (Guo et al. 2017; Walsh et al. 2017). For integrated cross-condition analysis, a total of 73 samples were selected, comprising 23 PCOS samples, 22 OA samples, and 28 RA samples. The sample size was determined based on the availability of matched case-control samples within the selected datasets. Downloaded raw count matrices were imported into R (version 4.2.0) for preprocessing. Samples were tested to examine quality control to confirm the integrity and metadata of sequencing data. Samples were categorized into respective groups by selecting condition labels manually. To satisfy the criteria of input for downstream analysis DESeq2 package, raw read counts were transformed into numeric matrices and rounded on the basis of need. To maintain the analytical significance, genes with very low expression were filtered.

### Identification of common genes of PCOS and arthritis

The DESeq2 package was used to identify DEGs for each dataset. The analysis included normalization through estimation of size factors, dispersion estimation, fitting of the negative binomial generalized linear model, and application of the Wald test for final hypothesis testing. Pairwise analyses were conducted for PCOS vs. Control, RA vs. Normal, OA vs. Normal, RA vs. OA, PCOS vs. RA, and PCOS vs. OA. Genes with an adjusted *p*-value < 0.1 and |log_2_ fold change| > 0.5 were considered significant and differentially expressed. Volcano plots were generated using both Enhanced-Volcano and ggplot2 to visualize upregulated and downregulated genes (Devarbhavi et al. 2021).

### Functional enrichment and cross-condition comparative analysis

To identify biological associations between the DEGs of PCOS and arthritis, a functional enrichment analysis was conducted. Significantly upregulated genes from each comparison were converted from gene symbols to Entrez IDs using the *org.Hs.eg.db* annotation database (Hagenauer et al. 2024). Gene Ontology (GO) enrichment analysis for the biological process category was performed using the *clusterProfiler* package, along with Kyoto Encyclopedia of Genes and Genomes (KEGG) pathway enrichment analysis. Only pathways with adjusted *p*-values less than 0.05 were considered significant. The results were visualized using bar plots to highlight biological processes and common signaling pathways involved in each condition. Cross-condition comparisons were conducted to identify shared molecular pathways among the conditions. Enriched GO terms and KEGG pathways from different diseases were used to determine overlapping pathways. Venn diagrams were used to identify common GO pathways enriched in PCOS vs. RA, PCOS vs. OA, and RA vs. OA.

### Software tools and Packages

R version 4.2+ was used for performing the bioinformatic analyses. The DESeq2 package was used for differential gene expression analysis; EnhancedVolcano and ggplot2 were used for visualization of DEGs; and the clusterProfiler package was used for functional enrichment analysis. The *org.Hs.eg.db* database was used for gene annotation, and Venn diagrams were generated to support cross-comparison studies between the diseases.

## Results

### Differential gene expression analysis

To evaluate the molecular alterations across PCOS and arthritis (RA and OA), transcriptomic profiles were compared to identify genes with significant expression differences. Volcano plots were generated, with the x-axis representing the log_2_ fold change and the y-axis showing the –log_10_ adjusted *p*-values, to visualize the distribution of DEGs.

**Figure 1:**
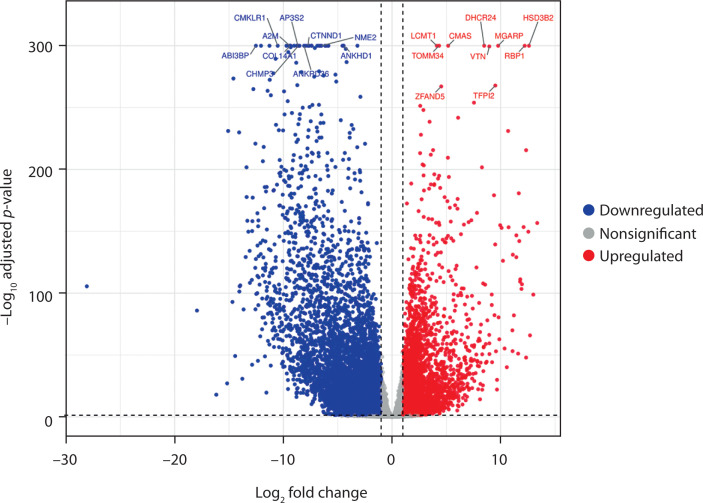
Volcano plot displays the differentially expressed genes in polycystic ovary syndrome (PCOS) versus osteoarthritis (OA) samples. The x-axis represents the log_2_ fold change in gene expression, and the y-axis shows the –log_10_ adjusted *p*-value. Significantly upregulated genes in PCOS (log_2_ FC > 1; adjusted *p* < 0.05) appear in red, while significantly downregulated genes in PCOS (active in OA) appear in blue (log_2_ FC < –1; adjusted *p* < 0.05). Nonsignificant genes are shown in gray. A total of 3,567 genes were upregulated and 6,325 were downregulated. Key differentially expressed genes are labeled

In the comparative analysis between PCOS and OA, a total of 9,892 DEGs were identified, of which 3,567 were upregulated and 6,325 were downregulated. *TOMM34, CMAS, DHCR2*, and *RBP1* were among the key genes that were highly expressed (Figure 1). The consistency of expressed genes across diseases indicates the presence of common molecular pathways.

**Figure 2:**
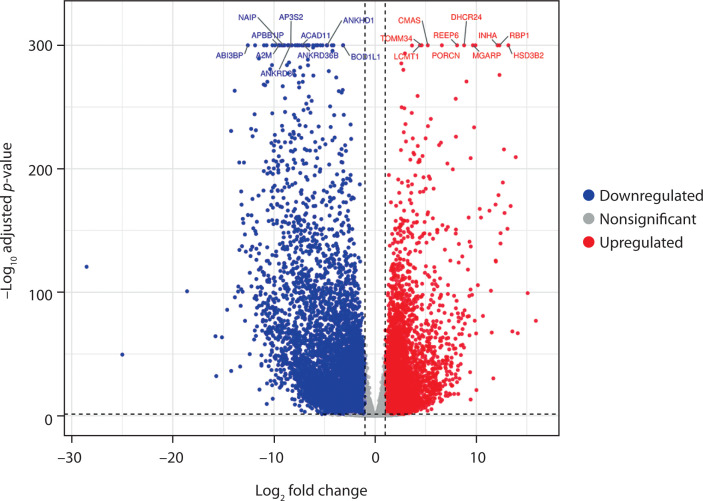
Volcano plot shows differentially expressed genes between polycystic ovary syndrome (PCOS) and rheumatoid arthritis (RA) samples. The x-axis indicates the log_2_ fold change, and the y-axis represents the –log_10_ adjusted *p*-value. Genes significantly upregulated in PCOS (log_2_ FC > 1; adjusted *p* < 0.05) are shown in red, while significantly downregulated genes (log_2_ FC < –1; adjusted *p* < 0.05) are shown in blue. Nonsignificant genes are displayed in gray. In total, 4,271 genes were upregulated and 6,221 were downregulated in PCOS compared with RA. Key differentially expressed genes are labeled

Similarly, the volcano plot for PCOS and RA revealed 10,492 DEGs, with 4,271 upregulated and 6,221 downregulated genes. The plot clearly separated genes based on their direction of regulation, and the main dysregulated genes included *CMAS, DHCR24, HSD3B2, INHA, NAIP, LCMT1*, and *A2M* (Figure 2). These findings suggest the presence of correlated pathways in both conditions and highlight the potential involvement of immune mechanisms in PCOS pathophysiology, along with reproductive dysregulation.

### Overlapping gene ontology analysis

**Figure 3:**
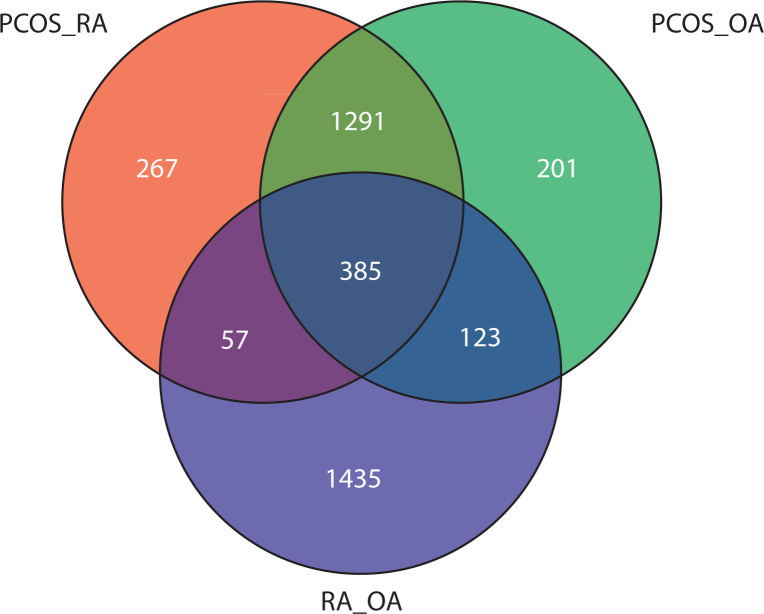
Venn diagram illustrates the overlap of enriched Gene Ontology (GO) pathways identified across three pairwise comparisons: polycystic ovary syndrome (PCOS) vs. rheumatoid arthritis (RA) (PCOS_RA), PCOS vs. osteoarthritis (OA) (PCOS_OA), and RA vs. OA (RA_OA). Each circle includes uniquely enriched pathways, while 385 pathways are commonly enriched across all three conditions

To gain deeper insight into the functional pathways associated with the DEGs, GO enrichment analysis was performed. Using the Venn tool, overlapping GO pathways among PCOS vs. RA, PCOS vs. OA, and RA vs. OA comparisons were identified. Figure 3 shows that 267 GO pathways overlap between PCOS and RA. OA and PCOS share 201 common pathways, while 123 GO pathways were found to be common between RA and OA. The Venn analysis also revealed that 57 GO pathways were enriched across all three conditions, indicating consistent dysregulation of biological processes among them.

### Kyoto Encyclopedia of Genes and Genomes (KEGG) pathway enrichment analysis

**Figure 4:**
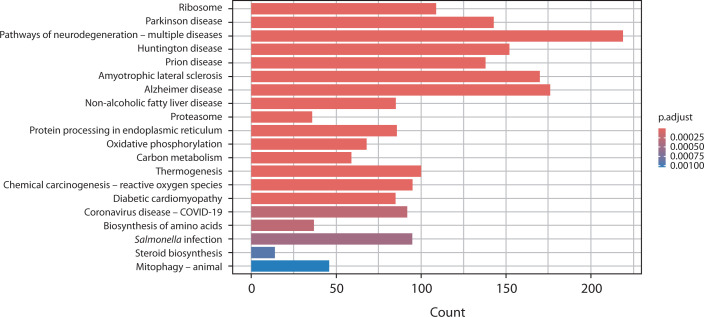
Bar chart presents the KEGG pathway enrichment analysis comparing gene expression between polycystic ovary syndrome and osteoarthritis. The x-axis shows the number of genes (count) associated with each pathway, and the y-axis lists the enriched KEGG pathways. Bars are color-coded according to the adjusted *p*-value (p.adjust), with darker red indicating stronger statistical significance

The KEGG database was used to analyze the biological and signaling pathways associated with the DEGs. In Figure 4, the PCOS vs. OA comparison highlights key pathways such as steroid biosynthesis, proteasome activity, and protein processing in the endoplasmic reticulum, along with mitochondrial-related pathways including oxidative phosphorylation, thermogenesis, and mitophagy. Neurodegeneration-related pathways and metabolic pathways such as carbon metabolism and the biosynthesis of amino acids were also found to be perturbed.

**Figure 5:**
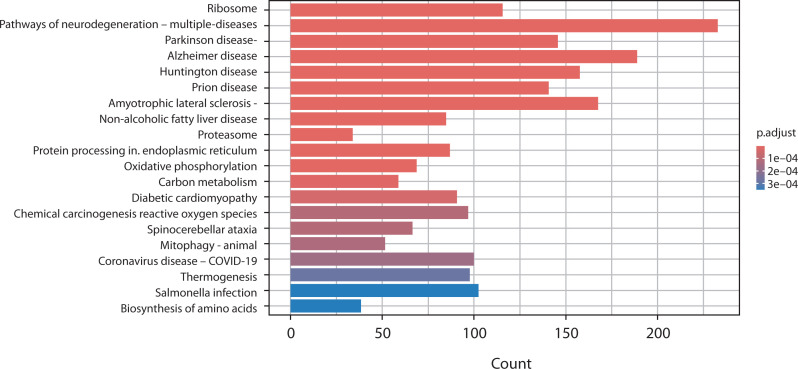
Bar chart presents the KEGG pathway enrichment analysis comparing gene expression between polycystic ovary syndrome and rheumatoid arthritis. The x-axis represents the number of genes (count) involved in each pathway, and the y-axis lists the enriched KEGG pathways. Bars are colored according to the adjusted *p*-value (p.adjust), with darker red indicating stronger statistical significance

In the comparison between PCOS and RA, several pathways were significantly enriched (adjusted *p*-value < 0.0001). Prominent pathways included proteasome activity, oxidative phosphorylation, and carbon metabolism, as well as immune-related pathways such as *Salmonella* infection, coronavirus pathways, and chemical carcinogenesis involving reactive oxygen species. Similar to OA, neurodegenerative pathways and amino acid biosynthesis pathways were observed, indicating underlying metabolic changes (Figure 5).

Furthermore, the overlapping nature of biological processes related to immune regulation, oxidative phosphorylation, mitochondrial function, metabolic regulation, and inflammatory signaling emphasizes the systemic hypothesis of PCOS, extending beyond its traditional classification as a reproductive disorder.

## Discussion

The present study examined the metabolic and systemic differences between PCOS, RA, and OA using a bioinformatic approach based on transcriptomic analysis. By performing enrichment analyses of DEGs, GO, and KEGG pathways, significant overlaps in molecular pathways and biological processes across the conditions were identified. This was further supported by the Venn tool, which used intersection analysis of enriched GO pathways to distinguish common and unique functional features.

The differential gene expression analysis showed significant transcriptional alterations when comparing PCOS to the autoimmune condition (RA) and the degenerative condition (OA). A total of 10,492 DEGs were identified between PCOS and RA (4,271 upregulated and 6,221 downregulated) and 9,892 between PCOS and OA (3,567 upregulated and 6,325 downregulated). *TOMM34, DHCR24, CMAS, RBP1*, and *HSD3B2* were key genes that exhibited significant expression across disease comparisons. The study’s hypothesis that PCOS affects biological processes beyond reproductive mechanisms is supported by these genes, which are involved in mitochondrial function, lipid metabolism, and immune regulation (Zhang et al. 2019; Siemers et al. 2023).

Further investigations revealed considerable overlap in gene pathways across the conditions. The Venn analysis of GO pathway enrichment highlighted a substantial overlap of 267 pathways between PCOS and RA, signifying common biological mechanisms related to immune regulation, including proteasome activity, oxidative stress, and cytokine signaling. These observations align with previous literature documenting elevated levels of pro-inflammatory cytokines such as TNF-α, IL-6, and CRP in women with PCOS, indicating a chronic low-grade inflammatory state (Sneha and Hiremath 2024; Vasyukova et al. 2023).

The overlap analysis of PCOS with OA revealed 201 shared pathways that affect mechanisms such as oxidative phosphorylation, protein degradation, and mitochondrial dysregulation. Although OA is typically characterized as a degenerative condition, this analysis emphasizes its involvement in a metabolic-inflammatory state. The shared steroid biosynthesis pathway between OA and PCOS further highlights the central role of hormonal imbalance in PCOS pathogenesis, which may also contribute to metabolic stress in OA (Zeber-Lubecka et al. 2023). Additionally, OA and RA were found to share 123 GO pathways associated with extracellular matrix remodeling, cartilage degradation, and inflammatory signaling. These results align with existing literature that describes shared mechanisms between autoimmune and degenerative joint diseases (Dan et al. 2024).

Importantly, 57 common GO pathways were identified across PCOS and arthritis (OA and RA). These pathways included those linked to endoplasmic reticulum (ER) stress, cellular stress responses, proteasomal degradation, mitophagy, and oxidative phosphorylation. The overlapping pathways suggest a consistent dysregulation of fundamental biological processes, potentially involving chronic inflammation, cellular stress, and metabolic dysfunction (Almanza et al. 2019; Chen et al. 2023). In particular, proteasome activity and ER stress are associated with protein quality control mechanisms, representing cellular responses to misfolded proteins and chronic inflammatory load. These findings are consistent with previous work highlighting the relationship between inflammation, mitochondrial dysfunction, and chronic systemic disease progression (Almanza et al. 2019).

Mitophagy and ER stress were notable common features identified across all three conditions. In PCOS, impaired mitophagy has been implicated in mitochondrial dysfunction and excess reactive oxygen species generation in ovarian tissue, resulting in insulin resistance and anovulation (Siemers et al. 2023). ER stress in granulosa cells can also disrupt steroidogenesis and follicular development (Koike et al. 2023). Similarly, dysregulated mitophagy and ER stress in synovial fibroblasts promote autoantigen presentation, hyperplasia, inflammatory cell survival, and cytokine production in RA (Bartok and Firestein 2010). In OA, defective mitophagy and ER stress in chondrocytes are associated with matrix degradation, aging-related joint degeneration, and cartilage loss (Cao et al. 2024). This shared immunometabolic etiology highlights the therapeutic potential of targeting cellular stress-response mechanisms in both inflammatory and endocrine disorders.

Finally, the transcriptomic findings support an association between PCOS and autoimmune-like features, emphasizing its complex and systemic nature. The disruption of common pathways involving immune, metabolic, and stress-related mechanisms demonstrates that PCOS extends beyond its conventional classification as a reproductive disease and may also represent a chronic inflammatory and degenerative condition. The study results provide a foundation for identifying novel therapeutic targets or biomarkers that may aid in early diagnosis or treatment. Further research is needed to confirm the shared pathways across disease groups through *in vitro* and *in vivo* experimentation.

## Conclusions

This research provides novel insights into the broader molecular landscape of PCOS by examining its transcriptomic relationship with autoimmune and degenerative disorders. Gene expression and functional pathway analyses have elucidated common cellular mechanisms, particularly those related to immune response, metabolic dysregulation, and endocrine signaling. The observed overlapping gene expression patterns indicate an association between PCOS and systemic inflammation, supporting the hypothesis of broader multisystem involvement in PCOS. These findings highlight relevant biomarkers and therapeutic targets; however, further studies are needed to translate these insights into diagnostic and treatment strategies.
